# Evolution of Bariatric Surgery in Italy in the Last 11 Years: Data from the SICOB Yearly National Survey

**DOI:** 10.1007/s11695-022-06435-9

**Published:** 2023-01-24

**Authors:** Paolo Gentileschi, Bruno Sensi, Leandro Siragusa, Roberto Sorge, Eliana Rispoli, Luigi Angrisani, Elisa Galfrascoli, Emanuela Bianciardi, Maria Paola Giusti, Maurizio De Luca, Marco Antonio Zappa, SICOB Survey Collaborative Group, Claudio Arcudi, Alessandro Balani, Rosario Bellini, Domenico Benavoli, Giovanna Berardi, Giovanni Casella, Nicola Basso, Maria Rosaria Cerbone, Nicola Di Lorenzo, Enrico Facchiano, Mirto Foletto, Pietro Forestieri, Diego Foschi, Ilenia Grandone, Marcello Lucchese, Emilio Manno, Mario Musella, Giuseppe Navarra, Stefano Olmi, Luigi Piazza, Vincenzo PIlone, Marco Raffaelli, Giuliano Sarro, Alberto Zaccaroni

**Affiliations:** 1 Department of Surgery, University of Rome Tor Vergata, Rome, Italy; 2 Bariatric and Metabolic Surgery Unit, San Carlo Di Nancy Hospital, Rome, Italy; 3 Department of Biostatistics, Policlinico Tor Vergata University, Rome, Italy; 4 SoftItalia Consulting, Naples, Italy; 5 Public Health Department “Federico II” University of Naples, Naples, Italy; 6 Department of General Surgery, Fatebenefratelli Hospital, Milan, Italy; 7 Department of Systems Medicine, Psychiatric Chair, University of Rome Tor Vergata, 00133 Rome, Italy; 8 Chief Department of General and Metabolic Surgery, Rovigo Hospital, Rovigo, Italy

**Keywords:** Bariatric surgery, Obesity, RYGB, OAGB, Sleeve gastrectomy

## Abstract

**Background:**

Bariatric surgery (BS) is a relatively novel surgical field and is in continuous expansion and evolution.

**Purpose:**

Aim of this study was to report changes in Italian surgical practice in the last decade.

**Methods:**

The Società Italiana di Chirurgia dell’Obesità (SICOB) conducted annual surveys to cense activity of SICOB centers between 2011 and 2021. Primary outcome was to detect differences in frequency of performance of adjustable gastric banding (AGB), sleeve gastrectomy (SG), Roux-en-Y gastric bypass (RYGB), one anastomosis gastric bypass (OAGB), bilio-pancreatic diversion (BPD), and gastric plication (GP). Secondary outcome was to detect differences in performance of main non-malabsorptive procedures (AGB + SG) and overall bypass procedures (RYGB + OAGB). Geographical differences were also investigated.

**Results:**

Median response rate was 92%. AGB declined from 36% of procedures in 2011 to 5% in 2021 (*p* < 0.0001). SG increased from 30% in 2011 to 55% in 2021 (*p* < 0.0001). RYGB declined from 25 to 12% of procedures (*p* < 0.0001). OAGB rose from 0% of procedures in 2011 to 15% in 2021 (*p* < 0.0001). BPD underwent decrease from 6.2 to 0.2% in 2011 and 2021, respectively (*p* < 0.0001). Main non-malabsorptive procedures significantly decreased while overall bypass procedures remained stable. There were significant differences among regions in performance of SG, RYGB, and OAGB.

**Conclusions:**

BS in Italy evolved significantly during the past 10 years. AGB underwent a decline, as did BPD and GP which are disappearing and RYGB which is giving way to OAGB. The latter is rising and is the second most-performed procedure after SG which has been confirmed as the preferred procedure by Italian bariatric surgeons.

**Graphical Abstract:**

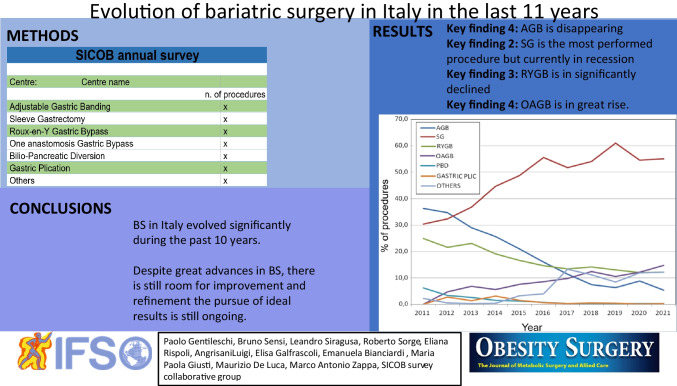

**Supplementary Information:**

The online version contains supplementary material available at 10.1007/s11695-022-06435-9.

## Introduction

Bariatric surgery (BS) is the single most important tool for the treatment of obesity-associated metabolic disease. Nonetheless, there is still ample room for improvement and new procedures or approaches are continuously being investigated. Roux-en-Y gastric bypass (RYGB) is the oldest and most studied BS procedure but its predominance has been challenged by sleeve gastrectomy (SG). New procedures such as one anastomosis gastric bypass (OAGB) and endoscopic approaches are being investigated while others such as bilio-pancreatic diversion (BPD) struggle to survive. In this scenario, it is important to keep track of changing trends in the BS community, in order to understand what is really going on outside of clinical trials. For this reason, the Italian national society for obesity surgery (Società Italiana di Chirurgia dell’OBesità — SICOB) has conducted an annual survey from 2011 through 2021.

## Methods

**Fig. 1:**
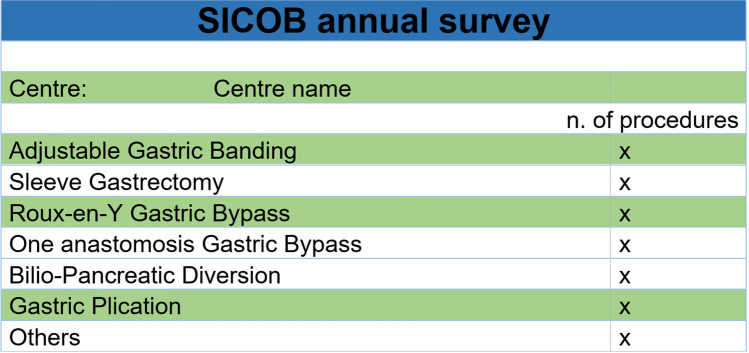
The SICOB annual survey (translated in English)

SICOB conducted annual surveys each year from 2011 (Fig. 1). The survey was sent to all SICOB centers. Until 2016, data was sent by e-mail to a centralized data management center. From 2016, data was retrospectively entered each year in an online registry by individual SICOB centers. Data regarding number, distribution, and activity of SICOB centers were collected, as were those regarding type of procedure performed. The study period was therefore between January 2011 and December 2021.

Centers were considered high volume when performing > 100 procedures per year and low volume if performing < 50 procedures per year.

### Aims and Outcomes

Aim of this study was to detect changes in Italian bariatric surgical practice. Primary outcomes were differences in frequency of performance of adjustable gastric banding (AGB), SG, RYGB (and its Italian variants), OAGB, BPD, GP, and others between 2011 and 2021. Secondary outcomes included changes in performance of main non-malabsorptive surgeries (AGB + SG) and overall bypass procedures (RYGB + OAGB) between 2011 and 2021.

Geographical differences in frequency of BS procedures performed were also recorded.

### Statistics

Data was collected in an excel database and were analyzed with IBM SPSS Statistics for Windows (Vers.27.0. Armonk, NY: IBM Corp.). Specific procedural data were normalized and expressed as percentage of total yearly procedures. To reduce possible extreme fluctuations, percentages were smoothened through the moving averages method. This method is used to smooth time series by averaging a fixed number of consecutive terms. The averaging “moves” over time, in that each data point of the series is sequentially included in the averaging, while the oldest data point in the span of the average is removed. To detect significant changes in procedure performance, ANOVA and Bonferroni tests were applied. Results were considered as statistically significant when *p* < 0.05.

## Results

A median of 92% of SICOB centers answered the survey each year. The years 2012 and 2017–2019 featured the worse SICOB center adherence to national registry data entry, which was under 80% (Table 1).

**Table 1:** Responders to the annual SICOB survey

Year	% of SICOB centers answering survey
2011	92%
2012	78%
2013	100%
2014	93%
2015	92%
2016	98%
2017	77%
2018	71%
2019	74%
2020	93%
2021	93%

### SICOB Centers

SICOB centers increased steadily from 91 in 2011 to 138 in 2021. Nonetheless, peak number of SICOB centers was achieved in 2019 (151). High-volume centers have represented almost 50% of SICOB centers for the whole study period. In 2021, 45% of centers were in the North of Italy, 21% and 24% in the center and South respectively, and the remaining 10% in the isles.

### Procedures

**Fig. 2:**
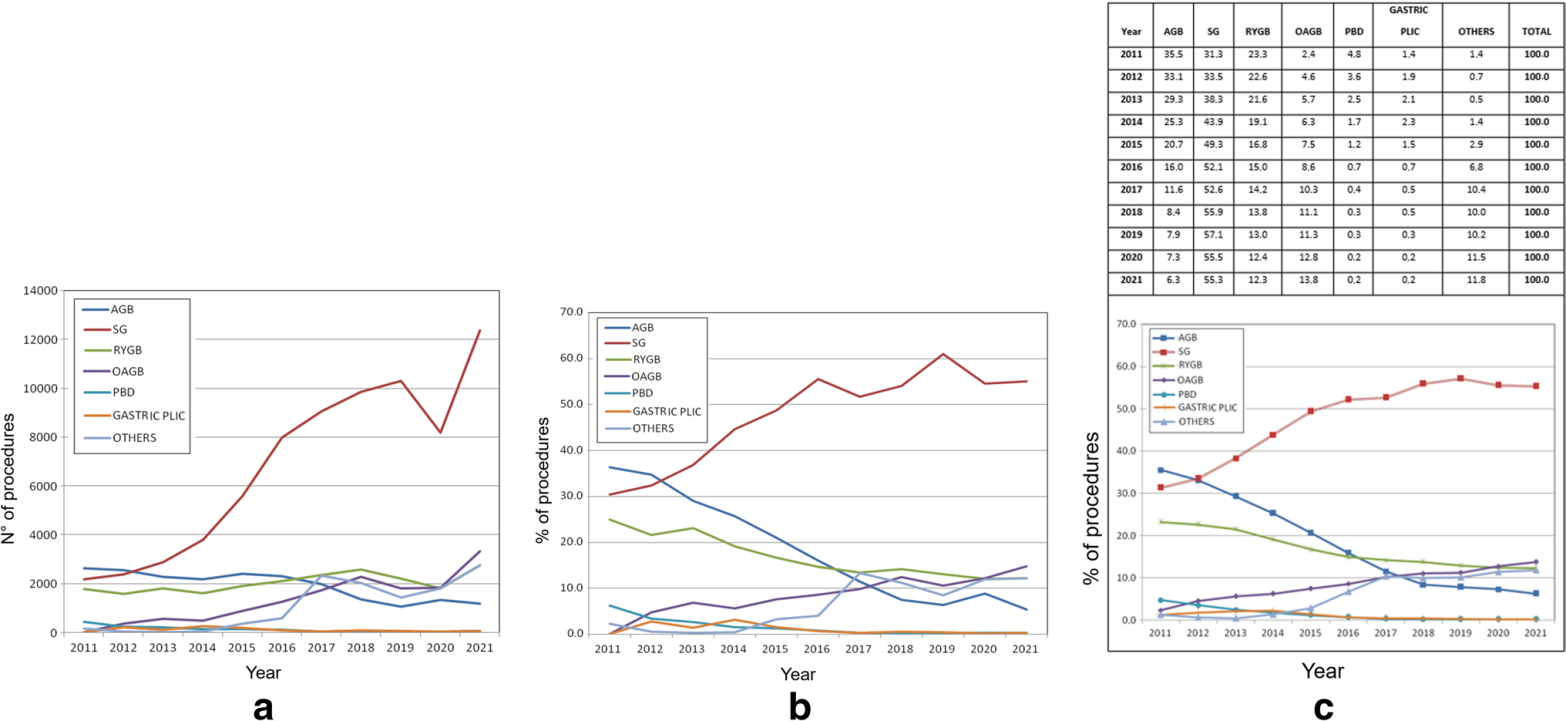
Yearly procedures 2011–2021. **A** Absolute numbers; **B** percentages; **C** moving averages

Procedures also increased steadily from 7645 in 2011 to 22,469 in 2021, despite a slight flexion in the curve in 2019 and 2020 (Fig. 2). Procedures performed included AGB, GP, SG, RYGB, OAGB, PBD, and others (including endoscopic procedures).

Performance of these procedures evolved significantly in these 11 years (Fig. 2 and Table 2).

**Table 2:** Yearly procedures 2011–2021% (*n*)

Year	AGB	SG	RYGB	OAGB	BPD	GP	Others	Total
2011	36.4% (2623)	30.3% (2188)	24.9% (1796)	0.0% (0)	6.2% (447)	0.0% (0)	2.2% (160)	100.0% (7214)
2012	34.7% (2556)	32.3% (2383)	21.6% (1593)	4.7% (348)	3.3% (246)	2.8% (203)	0.5% (38)	100.0% (7367)
2013	29.1% (2283)	36.8% (2889)	23.0% (1805)	6.9% (538)	2.6% (202)	1.4% (112)	0.3% (23)	100.0% (7852)
2014	25.6% (2182)	44.6% (3799)	19.1% (1628)	5.6% (477)	1.5% (124)	3.1% (268)	0.5% (40)	100.0% (8518)
2015	21.0% (2406)	48.7% (5594)	16.7% (1912)	7.6% (870)	1.2% (143)	1.6% (180)	3.3% (378)	100.0% (11,483)
2016	15.9% (2293)	55.5% (7976)	14.6% (2104)	8.6% (1239)	0.7% (101)	0.6% (82)	4.1% (586)	100.0% (14,381)
2017	11.3% (1988)	51.6% (9046)	13.5% (2361)	9.8% (1715)	0.2% (41)	0.2% (34)	13.3% (2335)	100.0% (17,529)
2018	7.4% (1351)	54.0% (9850)	14.2% (2581)	12.4 (2266)	0.2% (45)	0.5% (93)	11.2% (2040)	100.0% (18,226)
2019	6.3% (1065)	61.0% (102,919	13.1% (2205)	10.6% (1790)	0.3% (43)	0.4% (61)	8.4% (1425)	100.0% (16,880)
2020	8.8% (1325)	54.5% (8178)	12.1% (1814)	12.2% (1827)	0.2% (32)	0.2% (27)	12.0% (1801)	100.0% (15,004)
2021	5.3% (1191)	55.0% (12,359)	12.2% (2748)	14.8% (3325)	0.2% (53)	0.2% (46)	12.2% (2747)	100.0% (22,469)

*AGB*, adjustable gastric banding; *SG*, sleeve gastrectomy; *RYGB*, Roux-en-Y gastric bypass; *OAGB*, one anastomosis gastric bypass; *BPD*, biliopancreatic diversion; *GP*, gastric plication

#### AGB (Fig. S1A)

AGB was the most commonly performed procedure in 2011, with 36% of cases while it declined to barely 5% in 2021 (*p* < 0.0001). The decline was significant from 2011 through 2018 but particularly steep in the period 2014–2017, while reaching a plateau in 2018–2021.

#### SG (Fig. S1B)

SG represented 30% of procedures in 2011 and increased to become the leading procedure, with 55% in 2021 (*p* < 0.0001). The rise was significant from 2011 through 2016, particularly steep between 2012 and 2015, and reached stability in the years 2016–2021.

#### RYGB (Fig. S1C)

RYGB had a slow reduction in performance rates and overall procedures halved in the study time frame (from 25% in 2011 to 12% in 2021 respectively; *p* < 0.0001). The decline was significant from 2011 to 2016, most prominently in 2013–2015, and coming to a plateau in 2016–2021.

#### OAGB (Fig. S1D)

OAGB was never performed in 2011 and grew to represent 15% of procedures in 2021 (*p* < 0.0001). Its rise was significant from 2011 to 2020, particularly pronounced between 2014 and 2017, and is still growing.

#### BPD (Fig. S1E)

BPD accounted for 6.2% of BS in 2011 and 0.2% in 2021 (*p* < 0.0001). The recession was significant from 2011 to 2016, drastic between 2011 and 2014.

#### Gastric Plication (Fig. S1F)

Gastric plication was not performed in 2011, found acceptance and rose to 3.1% of operations in 2014 and then declined again to reach in 0.2% in 2017–2021 (*p* < 0.0001).

#### Other Procedures (Fig. S1G)

Other procedures were 2.2% in 2011 and 9.5% in 2021 (*p* < 0.0001). The increase began in 2013, was particularly rapid in 2015–2017, and halted between 2017 and 2021.

#### Main Non-malabsorptive Procedures (Fig. S1H)

During the study period, main non-malabsorptive procedures decreased from 66.7 to 60.3%. This decline was significant (*p* < 0.001), especially between 2015 and 2017.

#### Overall Bypass Procedures (Fig. S1I)

Overall bypass procedures remained stable throughout the study period ranging between 23.2% (in 2016) and 27.0% (in 2021 *p* = 0.197).

### Geographical Differences

Overall, AGB was performed uniformly in the country, representing between 8 and 10% of procedures. SG was the most-performed procedure in all regions but prevalence ranged from 39.3% in the isles to 70.1% in the North (*p* < 0.0001). There were significant differences in performance of RYGB which ranged from 11.2% in the North to 26.6% in the center (*p* < 0.0001). OAGB was performed in 7.9% of cases in the North and in up to 38.7% of cases in the isles (*p* < 0.0001).

## Discussion

This study reporting data from the SICOB annual surveys provides updated picture of real-world BS practice in Italy. The long-standing nature of these national surveys permits monitoring of BS activity in the country and their importance should not be underestimated [1]. Similar have been conducted in other countries in the past and frequently by IFSO worldwide, and represent precious testimony of BS practice evolution [2–8].

Data analysis has surfaced some clear trends. First, BS is still growing with an increasing number of centers performing bariatric operations and a threefold increase in overall yearly procedures performed during the study period. This data reflects the need for BS in Italy, in tight correlation with the ongoing obesity pandemic. In fact, obesity remains highly prevalent in Italy and it is associated with a high health burden and its associated costs [9, 10]. BS has been therefore increasingly recognized as the most effective and cost-effective treatment. Overall BS procedures have dropped only in 2019 and 2020; however, 2019 was the only year with a poor survey-response rate (only 73% of centers) and 2020 was plagued by the COVID-19 pandemic. Similar trends have been observed in other parts of the world such as the Asia–Pacific region [11].

In 2011, AGB was still the most commonly performed BS procedure. The last 10 years witnessed its gradual abandonment: AGB is now relegated to a very small percentage of BS procedures. This is certainly due to the now-established evidence of poor long-term outcomes of AGB, in relation to both high failure and complication rates (especially migration and erosion) [12]. This fact is in-line with preceding data and reflects a worldwide process [13, 14].

It is equally clear that AGB has been mostly replaced by SG, the adoption of which almost doubled. Its greater efficacy and lower complication rate determined its rise to become the most commonly performed BS procedure in Italy. This trend had been previously documented and SG appears to be the most-performed BS procedure today: in 2016, the IFSO worldwide survey confirmed predominance of SG in all regions except for South America [13, 14]. This was confirmed also at national levels, by several surveys in Europe (France, Spain, Germany) finding SG as the most-performed procedure and others in South America, for example with Brazil being quite refractory to SG uptake [4–7]. However, a new important trend has emerged here for the first time: the rise of SG has plateaued and has actually started a slow regression. This was unprecedented in previous studies. This trend is probably the result of tapering off of the initial enthusiasm, as time has made clear that SG has its own limitations including sub-optimal long-term efficacy and difficult-to-manage complications such as gastric leak and chronic GERD [15–19]. In fact, the absolute reduction in main non-malabsorptive procedures although small (6% between 2011 and 2021) was indeed significant. It is likely that some patients have been shifted from main non-malabsorptive procedures (i.e., SG in the last few years) to alternative “other procedures,” in particular to endoscopic approaches. In fact, in the last few years, other procedures consisted mainly of endoscopic approaches: unfortunately, data specifying other procedures was only collected after 2016; therefore, these assumptions cannot be verified for the whole study period.

Similarly to what happened for SG, “other procedures” also increased much until 2017 and then stopped below 10%. This may be connected to results of endoluminal procedures, which to-date are still less impacting than traditional BS [20]. A similar study in the USA found that use of intragastic balloon was reduced from 2018 [2].

Another clear trend is the slow but steady decline of RYGB, the application of which progressively reduced, reaching half its original employment by the end of the study period. This phenomenon appears to be common in other world regions as well, including North America and Europe but RYGB still represented 2125% of BS procedures in 2016 and this data was confirmed in 2018 [13, 14]. The current 12% rate is the lowest registered in the western world. This is in striking contrast with data from South America, where RYGB remains central and accounts for around 60% of BS activity and with Asia where RYGB was historically around 10% and remained so [13, 14]. RYGB remains considered by many the gold standard BS technique, due to its long-standing efficacy and safety and its superiority in the treatment of GERD [19]. However, RYGB is technically complex and long-term data have evidenced its fallibility [21].

In an opposite fashion, OAGB saw an unprecedented rise. OAGB is easier to fashion and randomized trials have yielded similar efficacy results to RYGB in the medium term [22, 23]. While no OAGB was performed in SICOB centers in 2011, it accounted for as many as 15% of BS in 2021. The rise in OAGB emerged also in the 2016 IFSO survey, especially for Europe, its exponentially increasing trend in Asia was registered in 2018 and while it remained rarely indicated in South America [13, 14]. However, the magnitude of the phenomenon reported here was unprecedented [13]. Even more remarkable is that OAGB is reported here to have surpassed RYGB, establishing itself as the second most-performed procedure after SG. It is likely that OAGB will continue to expand, possibly to the level of SG in years to come. Interestingly, despite existence of strong, mounting evidence on the short-, medium-, and long-term safety of OAGB, it is not endorsed until recently as primary BS in North America and can only be performed in the research setting. Therefore, in North America, data is extremely limited and OAGB has not known diffusion comparable to other world regions: even in accredited centers, it represented only 0.05% of BS and reported complication rates were (predictably?) much higher than generally reported [24].

In Italy, the success of OAGB explains how despite RYGB decline, there was no significant alteration in the performance of overall bypass procedures during the last decade. This may be interpreted as consistency in the indications for mild mal-absorptive procedures.

BPD was little used already in 2011 and is currently extinguishing, in line with worldwide trend, reflecting the idea of a mostly obsolete procedure, with high complication rate and to be reserved for very selected cases [13, 25]. Super-obese and refractory diabetic patients may still benefit from this procedure [25, 26].

It is noteworthy how there were significant differences in BS performance between Italian regions. The strong derangements seen in the south (39% SG and 38% OAGB) may probably be explained by the relatively low volume (10% of the total) and number of centers and therefore by the fact that the activity of few centers performing preferentially OAGB might have skew the percentages of the region as a whole. Differently, in the North, we see another trend that might be preoccupying in a way: there are a very large number of centers, performing a very large number of procedures but most of these are the technically less challenging AGB and SG (almost 80% of the total) while RYGB and OAGB account for a very limited percentage of procedures. This might reflect poor centralization and the existence of many poorly specialized centers performing technically convenient operations rather than giving patient-centered indications, yet this last assumption is purely speculative. Similar regionalized trends have been documented in the neighboring Switzerland, with SG being preferentially performed in Italian- and German-speaking areas rather than the French-speaking ones [3]. Nonetheless, this has been difficult to explain as in all three countries, SG appears to be the most-performed procedure, contrarily to Switzerland where RYGB represents the majority [3, 4, 7].

This study suggests how BS is in continuous refinement and evolution and allowed for the observation of some novel trends and phenomena.

The main limitations of this study are related to the method of data collection (self-reported email or online survey), the absence of patient outcomes, and the lack of distinction between primary and secondary bariatric procedures. This latter could be very meaningful in a future perspective, especially as other similar studies have reported a very consistent increase in secondary BS [2].

## Conclusions

BS is a constantly evolving field and the last 10 years of practice in Italy have seen profound changes. Absolute number of yearly procedures has increased more than threefold, testifying the rising consciousness of BS as a cost-effective method to contrast the obesity epidemic. AGB suffered a radical decline and was mainly substituted by SG, due to its more favorable efficacy/adverse event profile. Overall, main non-malabsorptive procedures slightly decreased. RYGB slowly declined in favor of OAGB, which represents the greatest and most impacting novelty due to its optimal results, low complication rate, and relative ease of performance. Indications for bypass procedures remained constant while BPD was largely abandoned. Significant inter-regional differences between geographical regions were also evidenced.

## Supplementary Information

Below is the link to the electronic supplementary material.

ESM 1 (PNG 91 kb)

High Resolution Image (TIF 95 kb)

ESM 2 (PNG 93 kb)

High Resolution Image (TIF 208 kb)

ESM 3 (PNG 94 kb)

High Resolution Image (TIF 211 kb)

ESM 4 (PNG 96 kb)

High Resolution Image (TIF 201 kb)

ESM 5 (PNG 91 kb)

High Resolution Image (TIF 209 kb)

ESM 6 (PNG 97 kb)

High Resolution Image (TIF 205 kb)

ESM 7 (PNG 94 kb)

High Resolution Image (TIF 135 kb)

ESM 8 (PNG 60 kb)

High Resolution Image (TIF 51 kb)

ESM 9 (PNG 57 kb)

High Resolution Image (TIF 51 kb)

## References

[1] Hopkins J, Ph D, Welbourn R (2016). The importance of national registries/databases in metabolic surgery : the UK experience. Surg Obes Relat Dis.

[2] Brethauer SA, Morton JM. American Society for Metabolic and Bariatric Surgery 2018 estimate of metabolic and bariatric procedures performed in the United States. Surg Obes Relat Dis. 2020. 10.1016/j.soard.2019.12.022 10.1016/j.soard.2019.12.022 32029370

[3] Gero D, Schneider MA, Suter M, Peterli R, Vonlanthen R, Turina M, Bueter M (2021). Sleeve gastrectomy or gastric bypass: a "post-code" lottery? A comprehensive national analysis of the utilization of bariatric surgery in Switzerland between 2011–2017. Surg Obes Relat Dis.

[4] Thaher O, Driouch J, Hukauf M, Glatz T, Croner RS, Stroh C (2022). Is development in bariatric surgery in Germany compatible with international standards? A review of 16 years of data. Updates Surg..

[5] Tonatto-filho AJ, Gallotti FM, Chedid MF, Grezzana-filho TDJM, Maria A, Vargas S (2019). Bariatric surgery in Brazilian public health system: the good, the bad and the ugly, or a long way to go YELLOW SIGN!. Arq Bras Cir DIg..

[6] Lecube A, Hollanda A De, Calañas A et al. Trends in bariatric surgery in Spain in the twenty-first century: baseline results and 1-month follow up of the RICIBA, a national registry. Obes Surg. 2015. 10.1007/s11695-015-2001-3 10.1007/s11695-015-2001-3 26661106

[7] Debs T, Petrucciani N, Kassir R et al. Trends of bariatric surgery in France during the last ten years. Analysis of 267 466 procedures from 2005 till 2014. Surg Obes Relat Dis. 2016. 10.1016/j.soard.2016.05.010 10.1016/j.soard.2016.05.010 27516221

[8] Laubner K, Prinz N, Brückel J, Serwas A, Altmeier M, Welp R, Krakow D, Groß F, Bollow E, Seufert J, Holl RW, DPV Initiative (2018). Comparative characteristics of patients with type 2 diabetes mellitus treated by bariatric surgery versus medical treatment: a multicentre analysis of 277,862 patients from the German/Austrian DPV database. Obes Surg.

[9] d'Errico M, Pavlova M, Spandonaro F (2022). The economic burden of obesity in Italy: a cost-of-illness study. Eur J Health Econ.

[10] Lauria L, Spinelli A, Buoncristiano M, Nardone P (2019). Decline of childhood overweight and obesity in Italy from 2008 to 2016: results from 5 rounds of the population-based surveillance system. BMC Public Health..

[11] Ohta M, Min S, Yosuke A, Wah S, Simon Y, Hung K (2021). Ten years of change in bariatric/metabolic surgery in the Asia-Pacific Region with COVID-19 pandemic: IFSO-APC National Reports. Obes Surg.

[12] Himpens J, Cadière G, Bazi M, Vouche M, Cadière B, Dapri G (2015). Long-term outcomes of laparoscopic adjustable gastric banding. Arch Surg.

[13] Angrisani L, Santonicola A, Iovino P, Ramos A, Shikora S, Kow L (2021). Bariatric surgery survey 2018: similarities and disparities among the 5 IFSO chapters. Obes Surg.

[14] Angrisani L, Santonicola A, Iovino P, Ramos A, Shikora S, Kow L, Bariatric surgery survey,  (2018). similarities and disparities among the 5 IFSO chapters. Obes Surg.

[15] Davrieux CF, Palermo M, Nedelcu M (2021). Reflux after sleeve gastrectomy: an update. J Laparoendosc Adv Surg Tech..

[16] Montuori M, Benavoli D, D'Ugo S, Di Benedetto L, Bianciardi E, Gaspari AL, Gentileschi P. Integrated approaches for the management of staple line leaks following sleeve gastrectomy. J Obes. 2017;2017:4703236. 10.1155/2017/4703236. 10.1155/2017/4703236 PMC5312046 28261497

[17] Matar R, Monzer N, Jaruvongvanich V, Abusaleh R, Vargas EJ, Maselli DB, Beran A, Kellogg T, Ghanem O, Abu Dayyeh BK (2021). Indications and outcomes of conversion of sleeve gastrectomy to roux-en-y gastric bypass: a systematic review and a meta-analysis. Obes Surg.

[18] Huh Y, Seob J, Lee S et al. Impacts of sleeve gastrectomy on gastroesophageal reflux disease in severely obese Korean patients. Asian J Surg. 2022. 10.1016/j.asjsur.2022.03.047 10.1016/j.asjsur.2022.03.047 35393223

[19] Genco A, Castagneto-Gissey L, Gualtieri L, Lucchese M, Leuratti L, Soricelli E, Casella G (2021). GORD and Barrett's oesophagus after bariatric procedures: multicentre prospective study. Br J Surg.

[20] Abu Dayyeh BK, Bazerbachi F, Vargas EJ, Sharaiha RZ, Thompson CC, Thaemert BC, Teixeira AF, Chapman CG, Kumbhari V, Ujiki MB, Ahrens J, Day C, Galvao Neto M, Zundel N, Wilson EB, MERIT Study Group (2022). Endoscopic sleeve gastroplasty for treatment of class 1 and 2 obesity (MERIT): a prospective, multicentre, randomised trial. Lancet..

[21] Christou NV, Look D, Maclean LD (2006). Weight gain after short- and long-limb gastric bypass in patients followed for longer than 10 years. Ann Surg.

[22] Robert M, Espalieu P, Pelascini E (2019). Efficacy and safety of one anastomosis gastric bypass versus Roux-en-Y gastric bypass for obesity (YOMEGA): a multicentre, randomised, open-label, non-inferiority trial. Lancet.

[23] Gentileschi P, Siragusa L, Alicata F, Campanelli M, Bellantone C, Musca T, Bianciardi E, Arcudi C, Benavoli D, Sensi B (2022). Nutritional status after Roux-En-Y (Rygb) and one anastomosis gastric bypass (Oagb) at 6-month follow-up: a comparative study. Nutrients..

[24] Jung JJ, Park AK, Hutter MM. The United States experience with one anastomosis gastric bypass at MBSAQIP‑accredited centers. Obes Surg. 2022;(0123456789). 10.1007/s11695-022-06002-2 10.1007/s11695-022-06002-2 36008649

[25] Topart PA, Becouarn G. Revision and reversal after biliopancreatic diversion for excessive side effects or ineffective weight loss: a review of the current literature on indications and procedures. Surg Obes Relat Dis. 2015:1–8. 10.1016/j.soard.2015.01.015 10.1016/j.soard.2015.01.015 25726366

[26] Skogar ML, Sundbom M (2017). Duodenal switch is superior to gastric bypass in patients with super obesity when evaluated with the bariatric analysis and reporting outcome system (BAROS). Obes Surg.

