# Supplementary figures and images for: Evolution of Bariatric Surgery in Italy in the Last 11 Years: Data from the SICOB Yearly National Survey
Source: Obes Surg. 2023 Jan 24;33(3):930–7. doi: 10.1007/s11695-022-06435-9 (PMC9871429; doi:10.1007/s11695-022-06435-9)

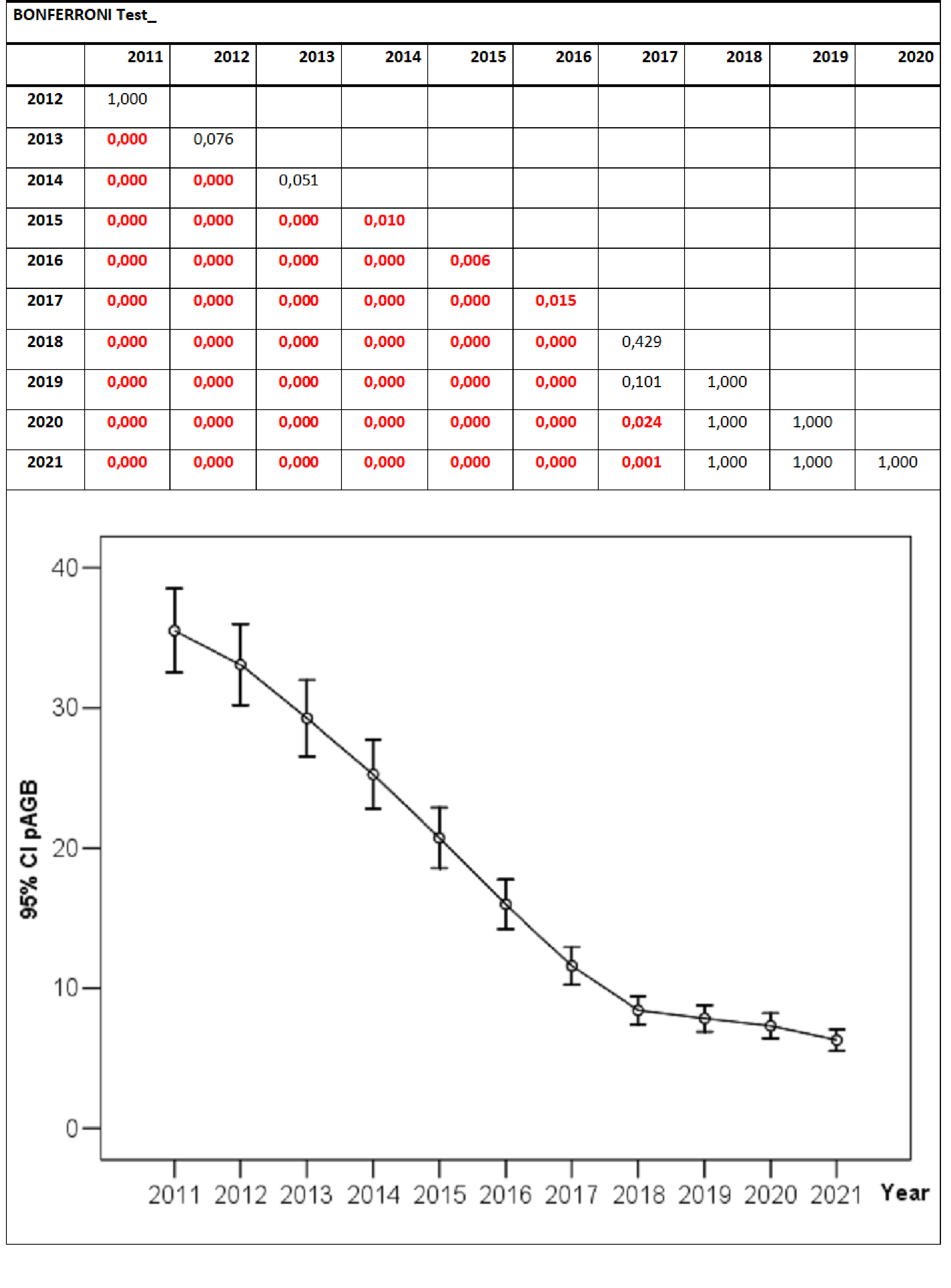

Supplement: Supplementary file 1 — (PNG 91 kb) [file 11695_2022_6435_Fig3_ESM.png]

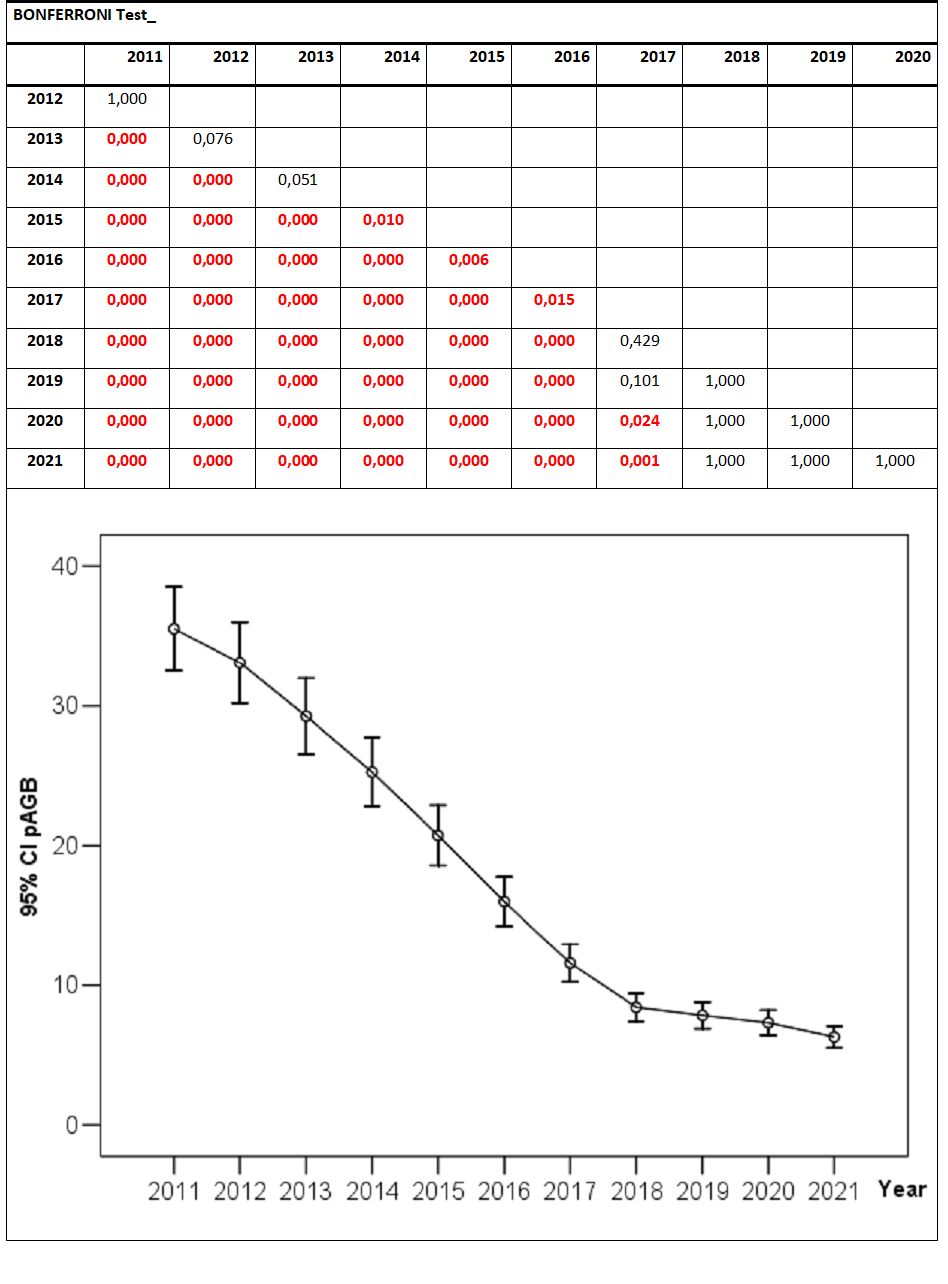

Supplement: Supplementary file 2 — High Resolution Image (TIF 95 kb) [file 11695_2022_6435_MOESM1_ESM.tif]

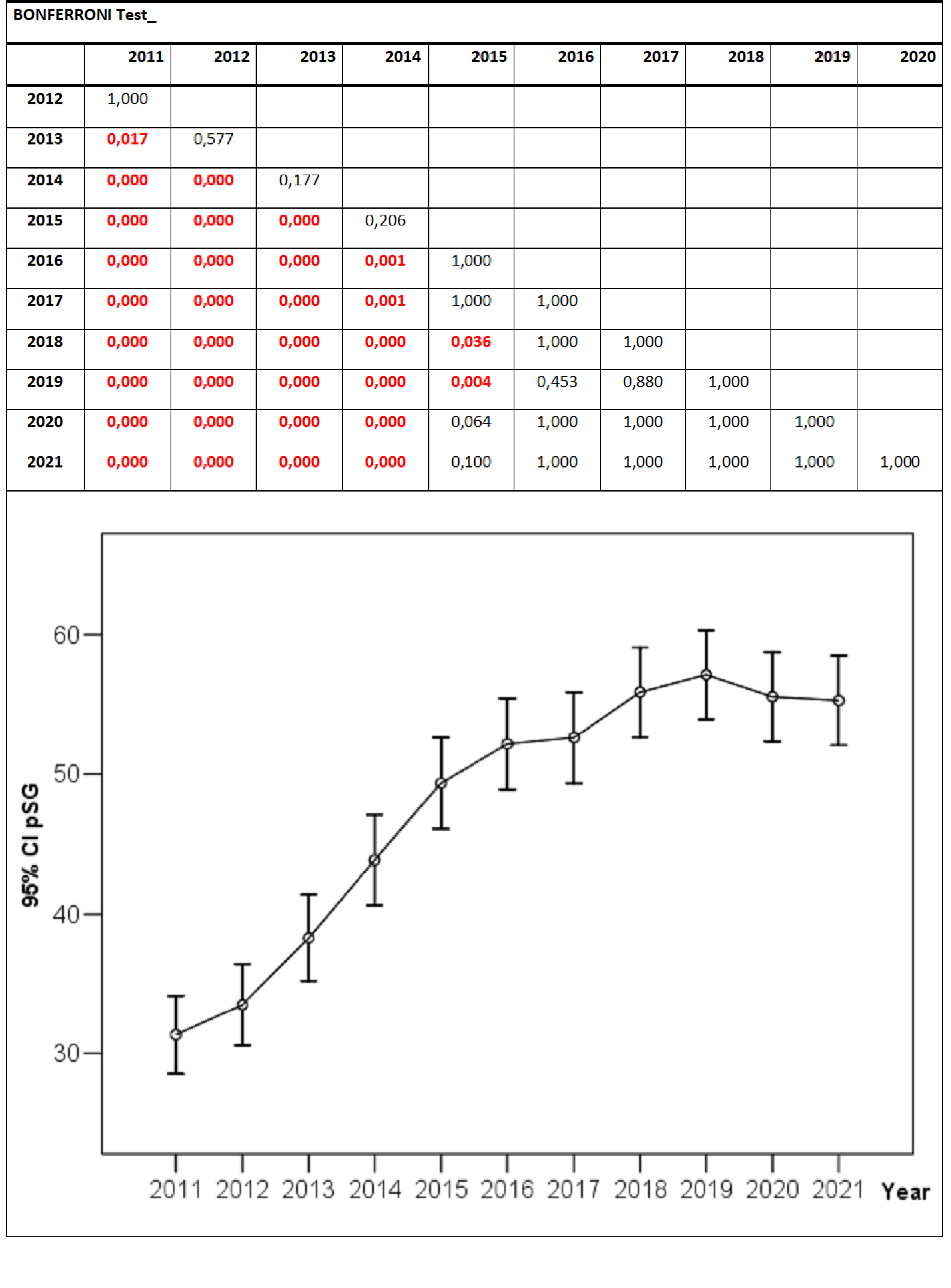

Supplement: Supplementary file 3 — (PNG 93 kb) [file 11695_2022_6435_Fig4_ESM.png]

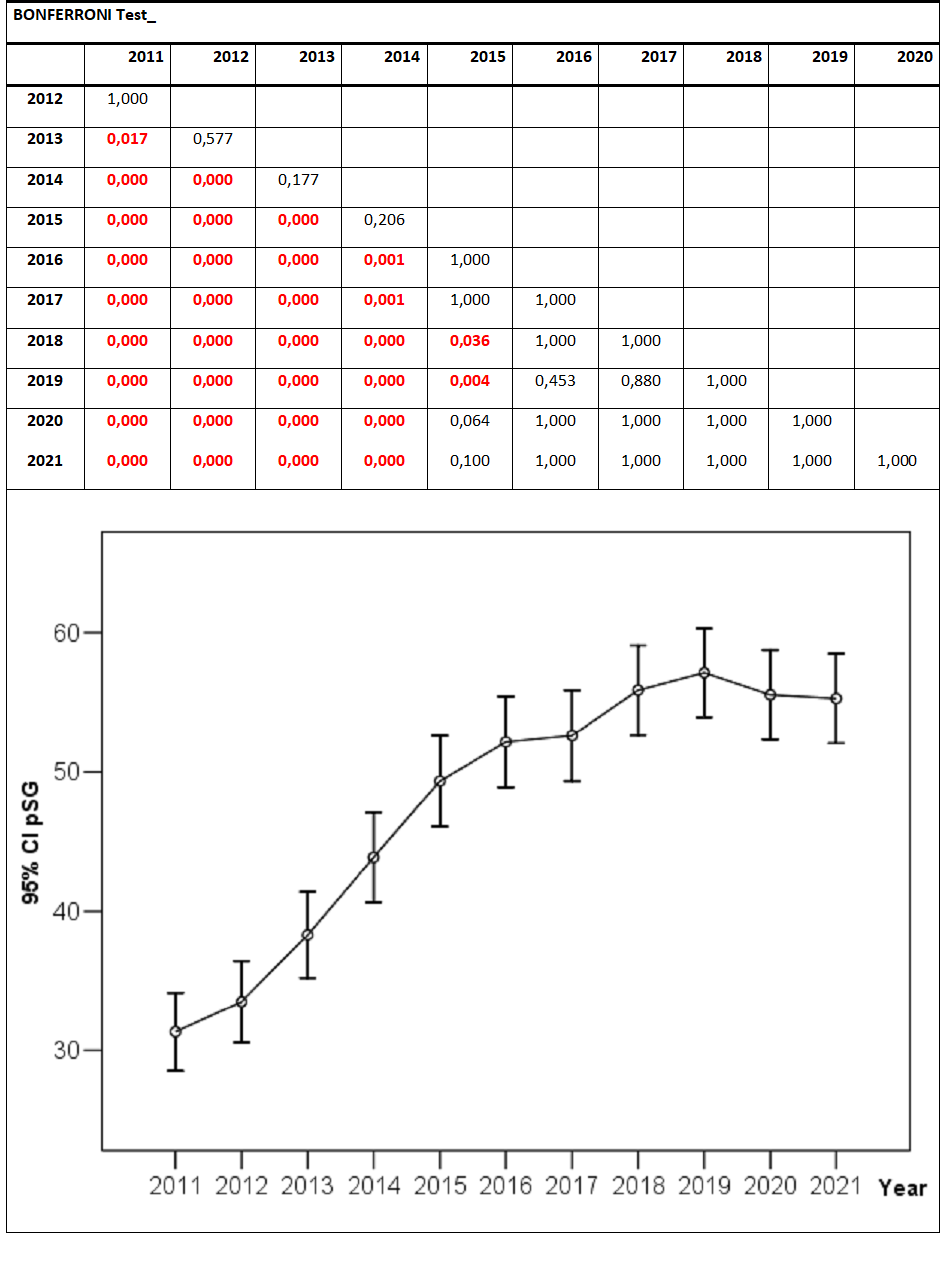

Supplement: Supplementary file 4 — High Resolution Image (TIF 208 kb) [file 11695_2022_6435_MOESM2_ESM.tif]

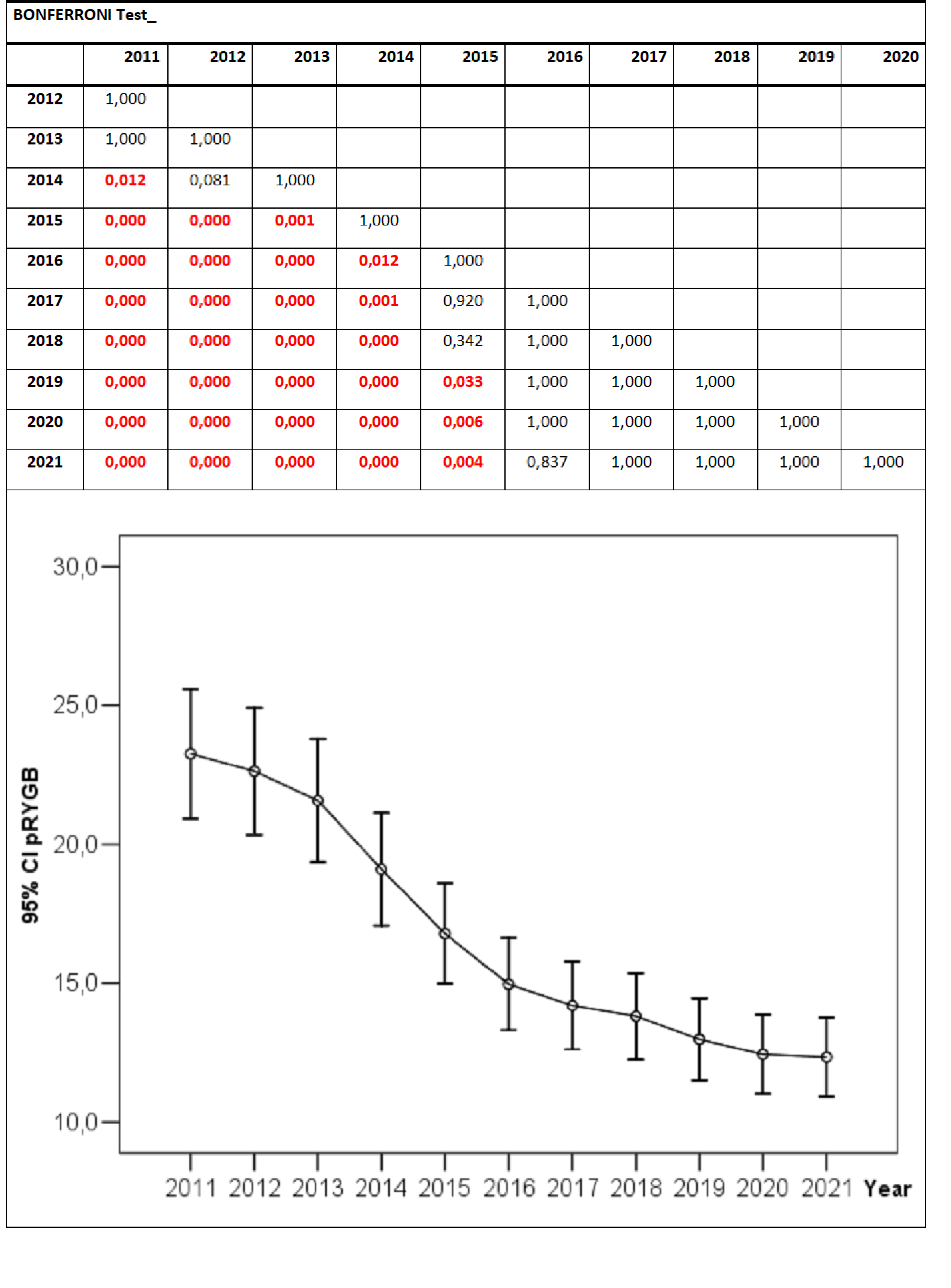

Supplement: Supplementary file 5 — (PNG 94 kb) [file 11695_2022_6435_Fig5_ESM.png]

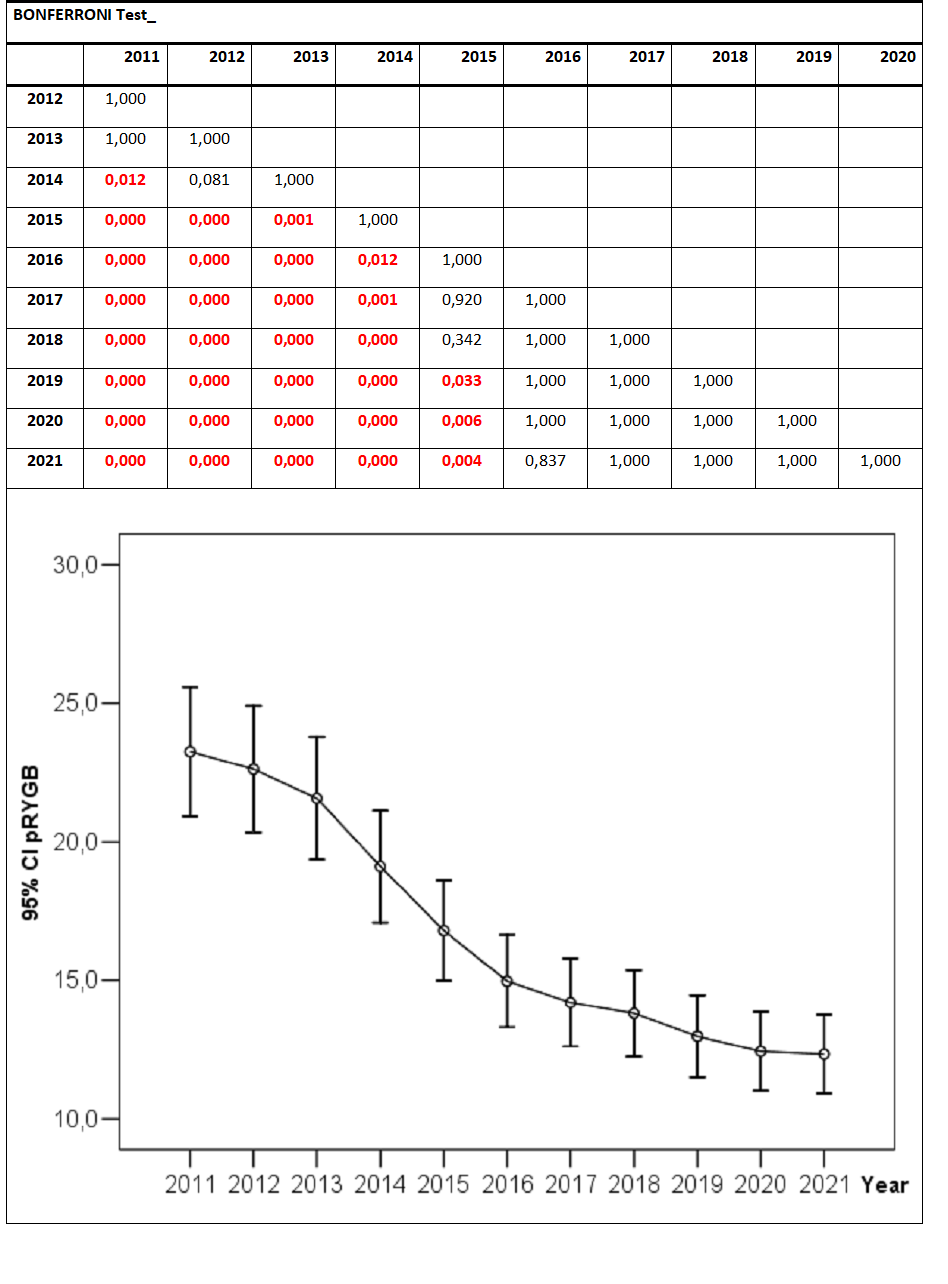

Supplement: Supplementary file 6 — High Resolution Image (TIF 211 kb) [file 11695_2022_6435_MOESM3_ESM.tif]

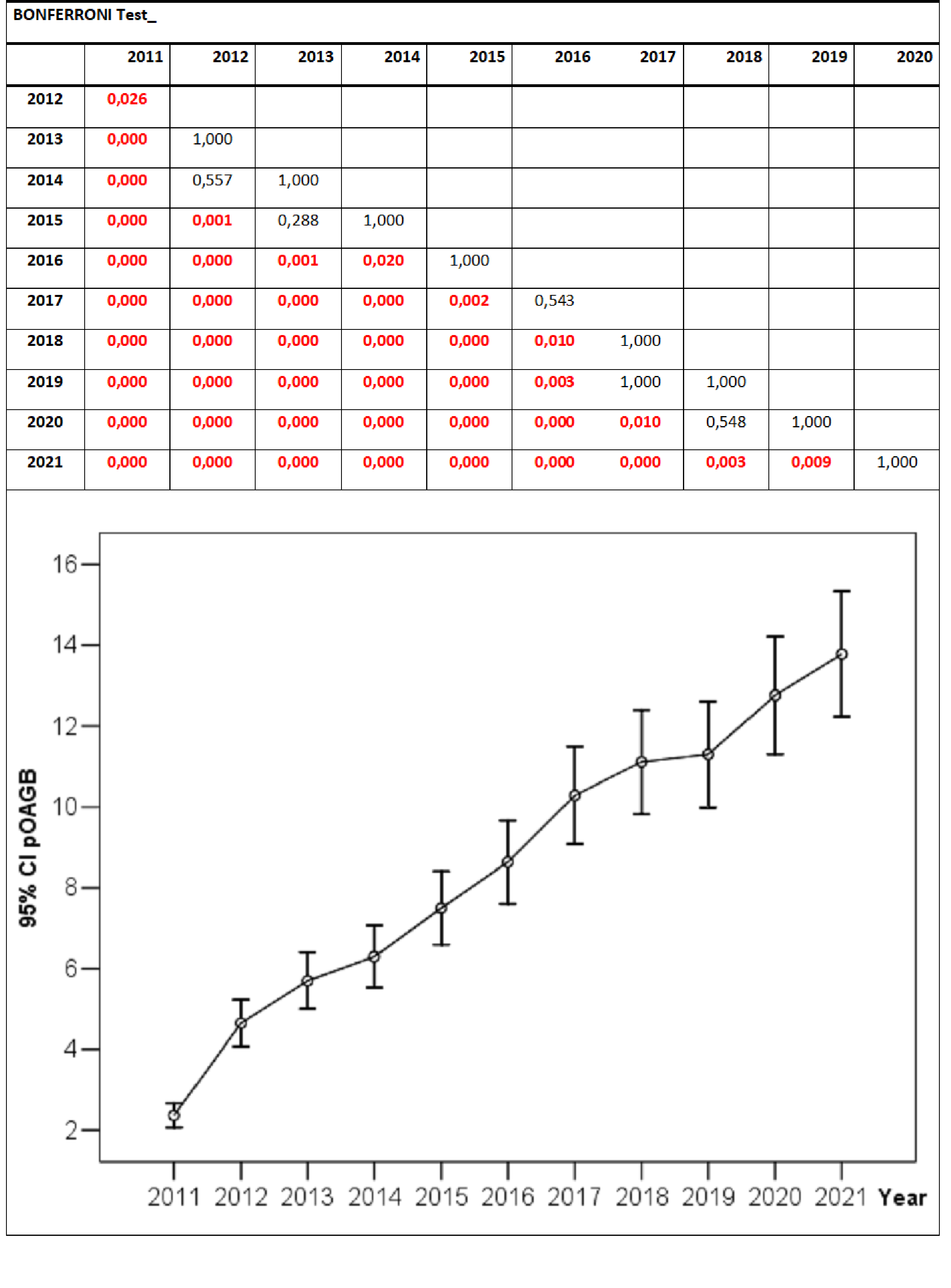

Supplement: Supplementary file 7 — (PNG 96 kb) [file 11695_2022_6435_Fig6_ESM.png]

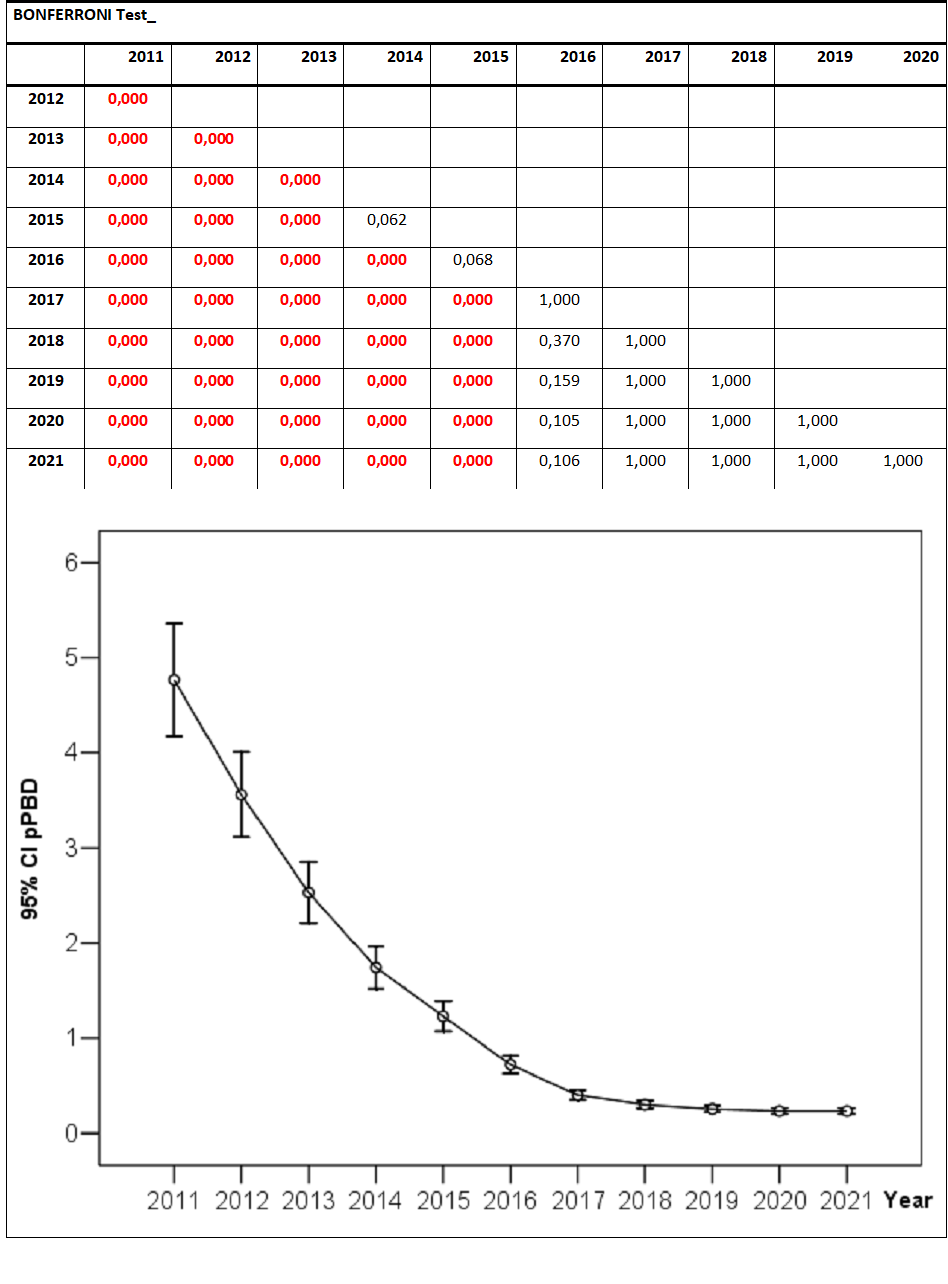

Supplement: Supplementary file 8 — High Resolution Image (TIF 201 kb) [file 11695_2022_6435_MOESM4_ESM.tif]

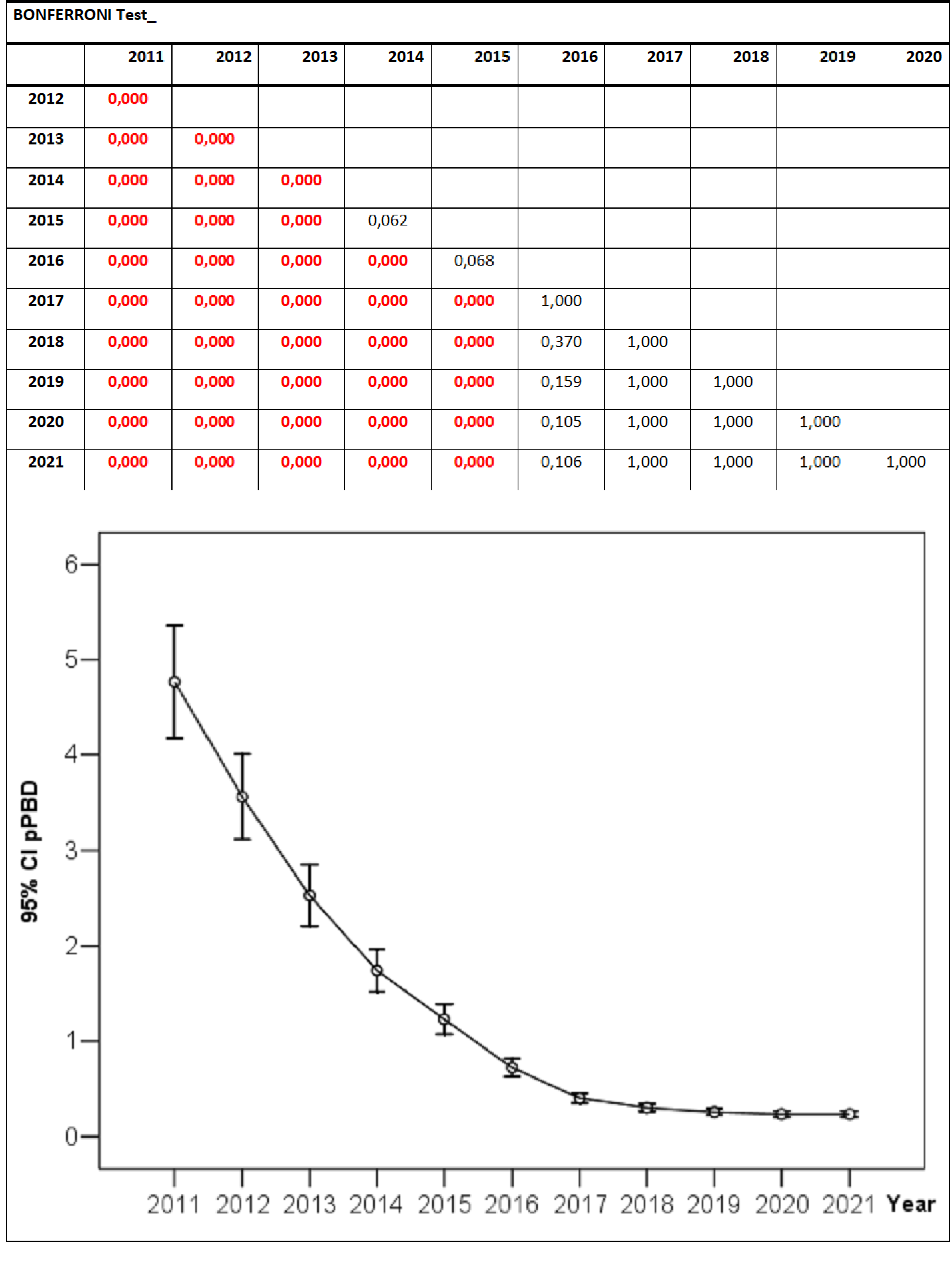

Supplement: Supplementary file 9 — (PNG 91 kb) [file 11695_2022_6435_Fig7_ESM.png]

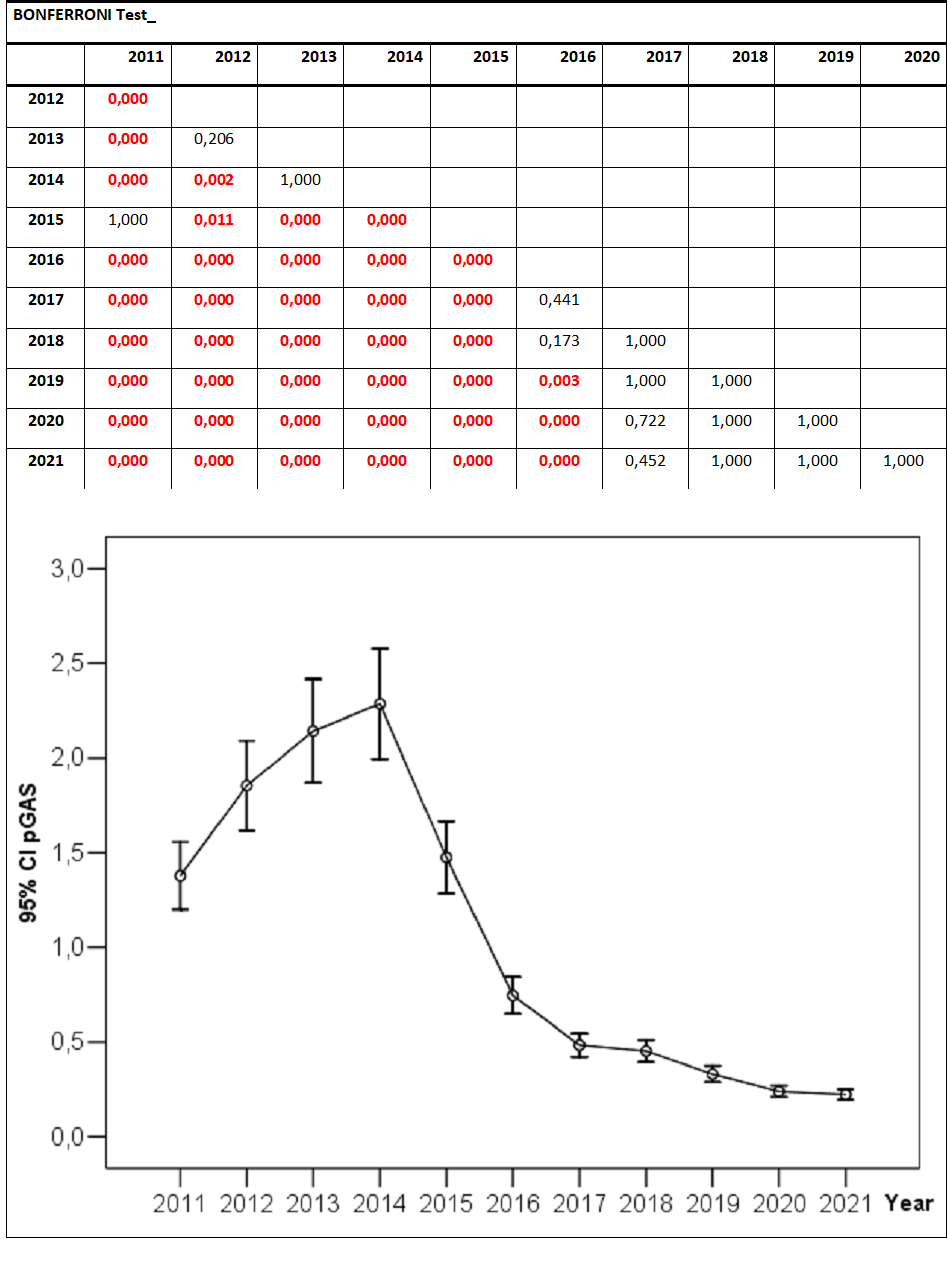

Supplement: Supplementary file 10 — High Resolution Image (TIF 209 kb) [file 11695_2022_6435_MOESM5_ESM.tif]

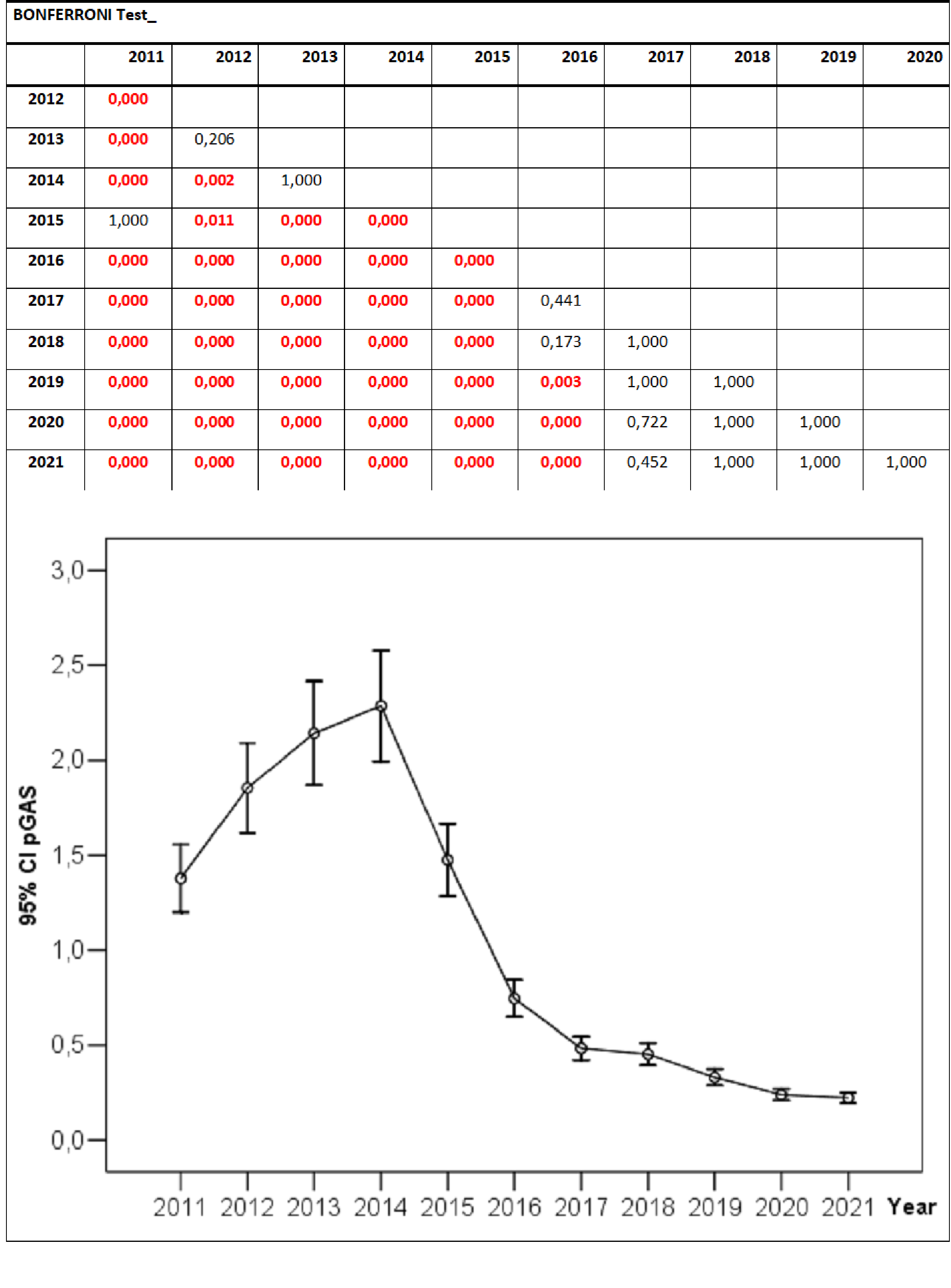

Supplement: Supplementary file 11 — (PNG 97 kb) [file 11695_2022_6435_Fig8_ESM.png]

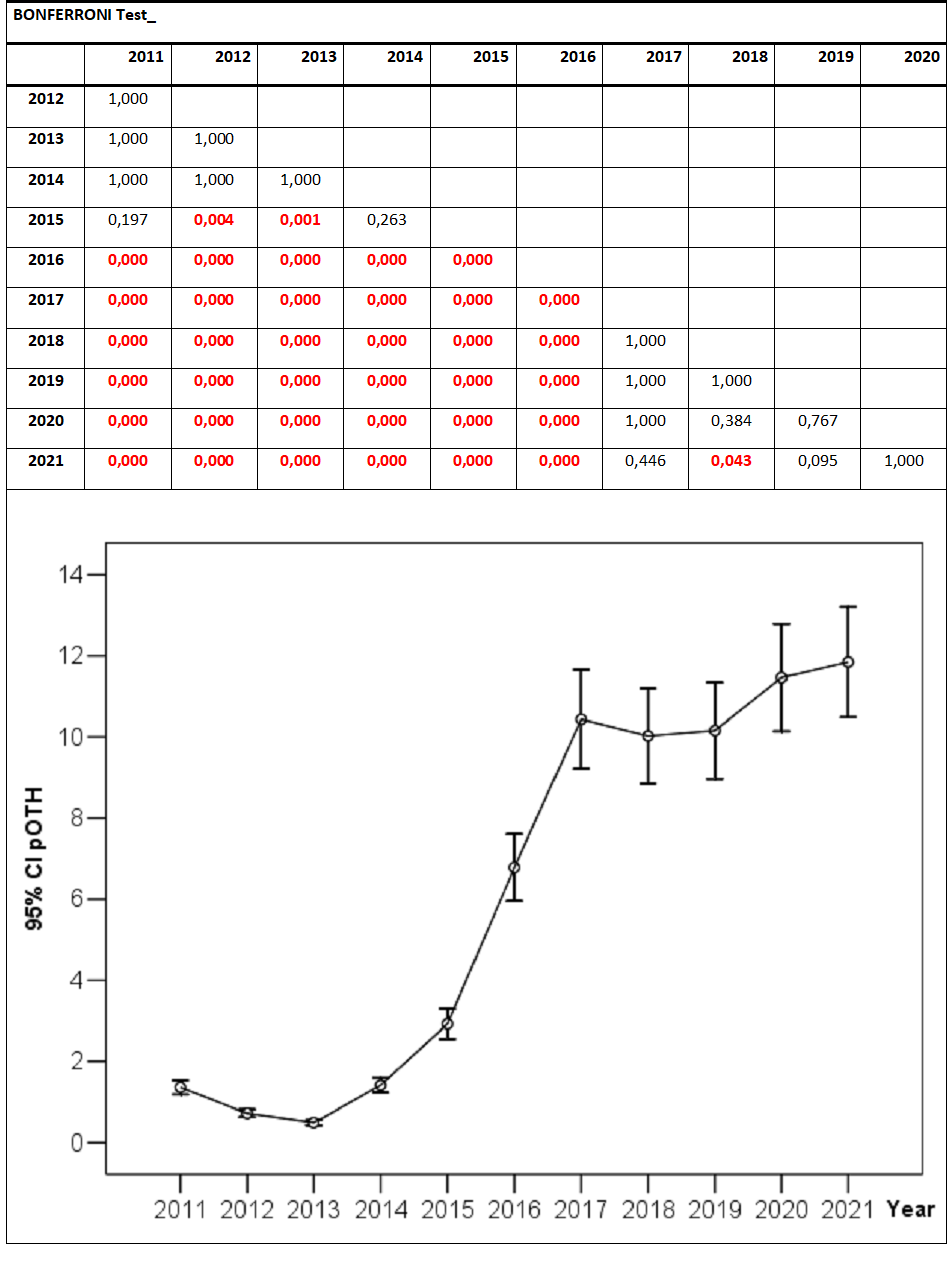

Supplement: Supplementary file 12 — High Resolution Image (TIF 205 kb) [file 11695_2022_6435_MOESM6_ESM.tif]

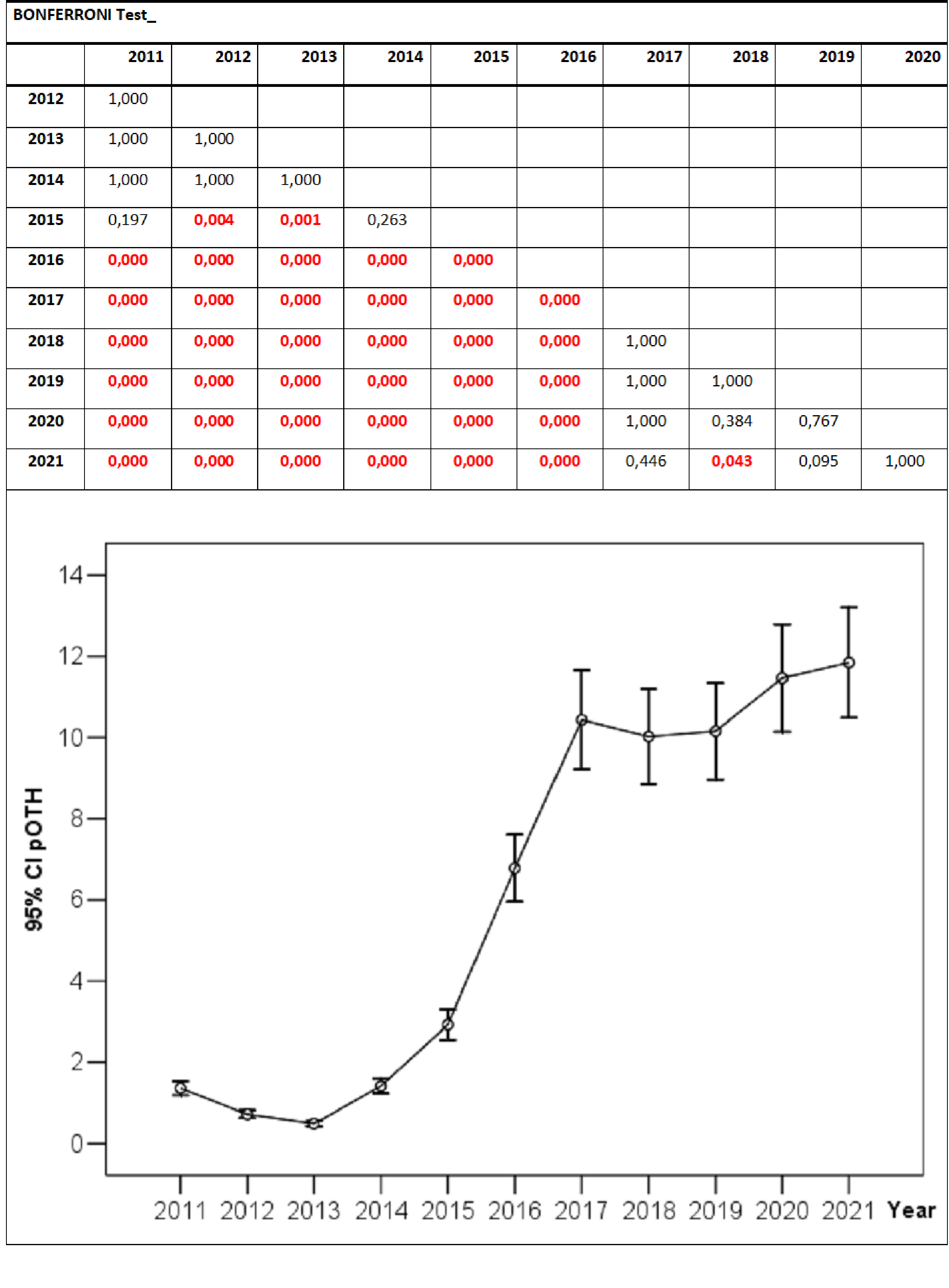

Supplement: Supplementary file 13 — (PNG 94 kb) [file 11695_2022_6435_Fig9_ESM.png]

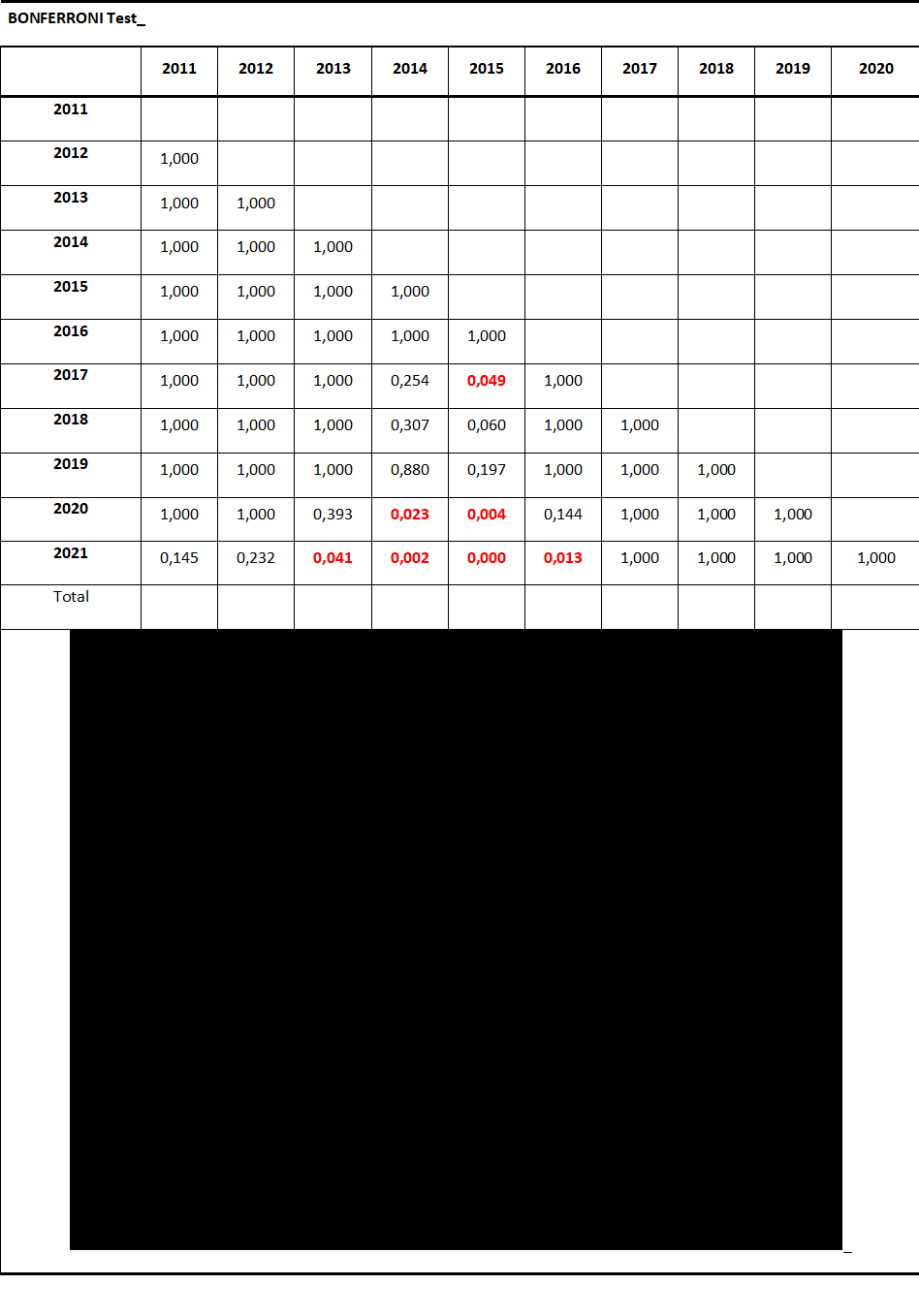

Supplement: Supplementary file 14 — High Resolution Image (TIF 135 kb) [file 11695_2022_6435_MOESM7_ESM.tif]

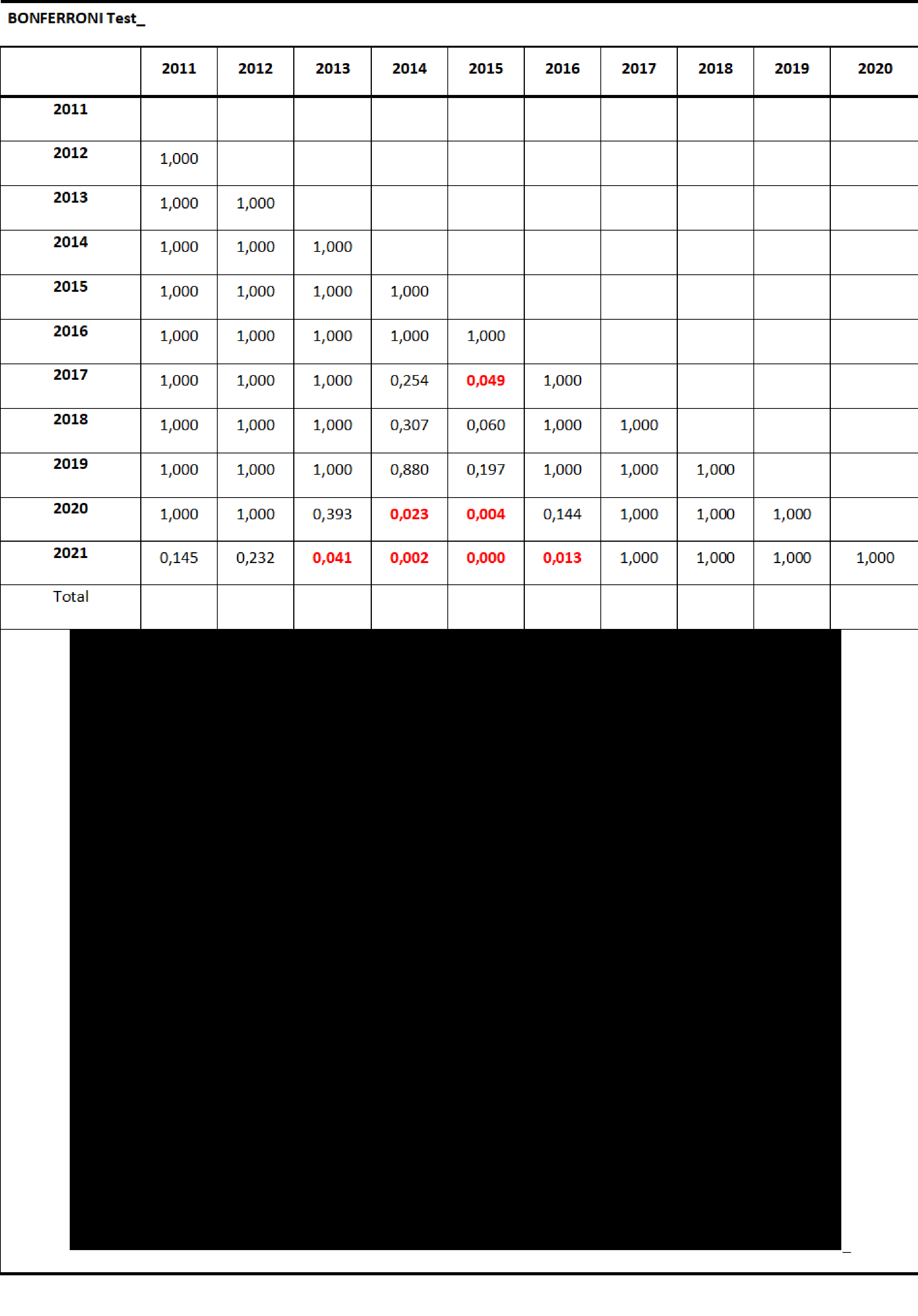

Supplement: Supplementary file 15 — (PNG 60 kb) [file 11695_2022_6435_Fig10_ESM.png]

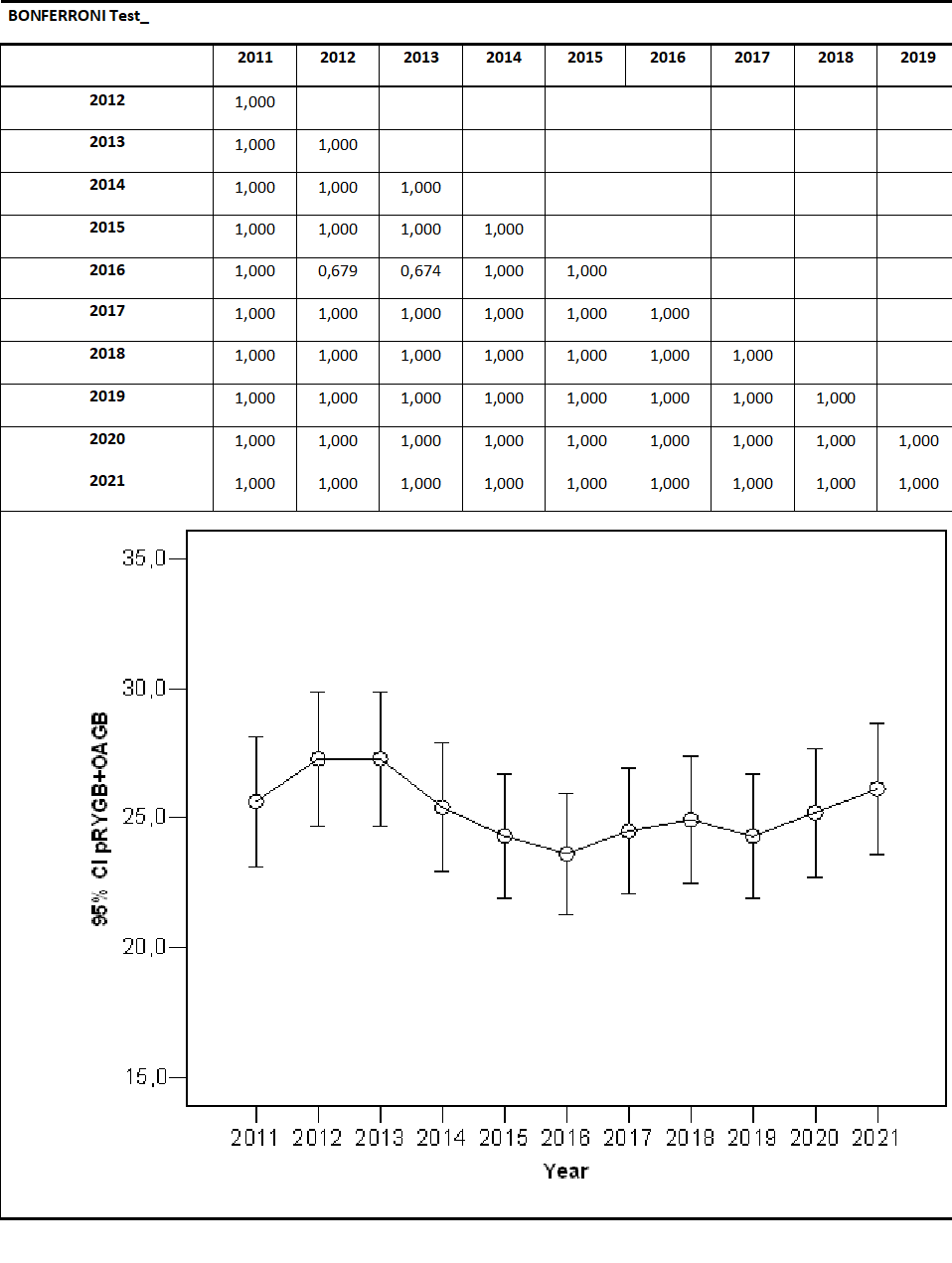

Supplement: Supplementary file 16 — High Resolution Image (TIF 51 kb) [file 11695_2022_6435_MOESM8_ESM.tif]

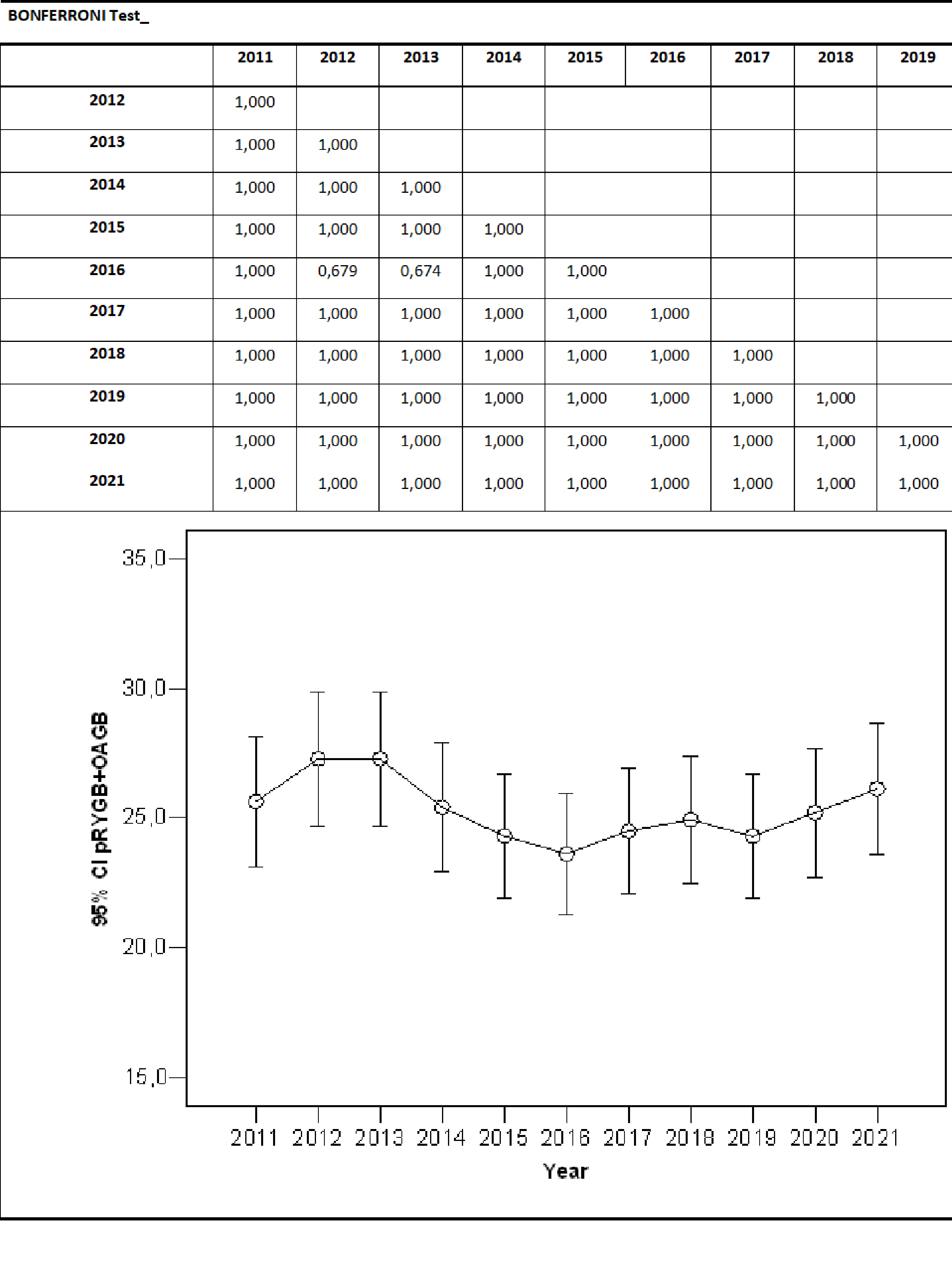

Supplement: Supplementary file 17 — (PNG 57 kb) [file 11695_2022_6435_Fig11_ESM.png]
